# Blue–Yellow VEP with Projector-Stimulation in *Glaucoma*

**DOI:** 10.1007/s00417-021-05473-w

**Published:** 2021-11-25

**Authors:** Laura Dussan Molinos, Cord Huchzermeyer, Robert Lämmer, Jan Kremers, Folkert K. Horn

**Affiliations:** Department of Ophthalmology and University Eye Hospital, Friedrich-Alexander University Erlangen-Nürnberg, Schwabachanlage 6, 91054 Erlangen, Germany

**Keywords:** Onset–offset VEP, Blue–yellow VEP, Glaucoma, Projector-stimulation

## Abstract

**Background and aim:**

In the past, increased latencies of the blue-on-yellow pattern visually evoked potentials (BY-VEP), which predominantly originate in the koniocellular pathway, have proven to be a sensitive biomarker for early glaucoma. However, a complex experimental setup based on an optical bench was necessary to obtain these measurements because computer screens lack sufficient temporal, spatial, spectral, and luminance resolution. Here, we evaluated the diagnostic value of a novel setup based on a commercially available video projector.

**Methods:**

BY-VEPs were recorded in 126 participants (42 healthy control participants, 12 patients with ocular hypertension, 17 with “preperimetric” glaucoma, and 55 with perimetric glaucoma). Stimuli were created with a video projector (DLP technology) by rear projection of a blue checkerboard pattern (460 nm) for 200 ms (onset) superimposed on a bright yellow background (574 nm), followed by an offset interval where only the background was active. Thus, predominantly S-cones were stimulated while L- and M-cone responses were suppressed by light adaptation. Times of stimulus onset to VEP onset-trough (N-peak time) and offset-peak (P-peak time) were analyzed after age-correction based on linear regression in the normal participants.

**Results:**

The resulting BY-VEPs were quite similar to those obtained in the past with the optical bench: pattern-onset generated a negative deflection of the VEP, whereas the offset-response was dominated by a positive component. N-peak times were significantly increased in glaucoma patients (preperimetric 136.1 ± 10 ms, *p* < 0.05; perimetric 153.1 ± 17.8 ms, *p* < 0.001) compared with normal participants (123.6 ± 7.7 ms). Furthermore, they were significantly correlated with disease severity as determined by visual field losses retinal nerve fiber thinning (Spearman R = –0.7, *p* < 0.001).

**Conclusions:**

Video projectors can be used to create optical stimuli with high temporal and spatial resolution, thus potentially enabling sophisticated electrophysiological measurements in clinical practice. BY-VEPs based on such a projector had a high diagnostic value for detection of early glaucoma.

Registration of study

Registration site: www.clinicaltrials.gov

Trial registration number: NCT00494923.


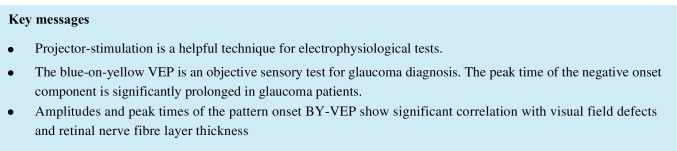


## Introduction

Chronic open-angle glaucoma is associated with the continuing loss of optic nerve fibers and subsequent loss of vision [1]. To reduce the risk of ongoing damage of the optic nerve, it is of utmost importance to detect glaucoma at its earliest stage and to monitor the progression of the disease continuously [2]. The diagnosis of chronic open-angle glaucoma has been based on the classic triad of increased intraocular pressure, cupping of the optic nerve head, and visual field defects [3, 4]. There are several procedures to detect the damage of the ganglion cells and the functional losses in glaucoma at an early stage [5]. Modern measurements include psychophysical techniques, including perimetry, imaging procedures, and electrophysiological measurements [6].

Visual evoked potentials (VEPs) are a tool to investigate the visual pathways up to the visual cortex [7]. Electro-encephalographic potentials provoked by visual stimulation are recorded from the visual cortex after passing the retinal and subcortical visual pathways [8, 9]. It has been shown that the VEP shows glaucoma induced changes when chromatic stimuli are applied [3]. When using isoluminant chromatic contrast VEP, the response may reflect the activity of the parvocellular and koniocellular pathways [10]. VEPs using red–green or blue–yellow stimuli proved to be sensitive to early glaucoma and were correlated with increasing functional and structural glaucomatous damage [11]. The blue-on-yellow pattern VEP [12] strongly stimulates the short-wavelength sensitive (koniocellular) pathway, which was found to be highly susceptible to glaucomatous damage[13 , 14, 15]. The study by Korth and co-workers [12] revealed that the blue-on-yellow pattern-VEP can be delayed by glaucoma. This earlier investigation used an optical bench for stimulation of the blue sensitive pathway with two monochromators in a Maxwellian view system. In these tests, a blue pattern light was superimposed on a yellow adaptation background using a vibrating mirror. When the mirror stopped the pattern was visible, when the mirror vibrated, the pattern was smeared over the background and thus not detectable resulting in an on- and offset of white–yellow bars without changing the time and space averaged luminance. Sensitivity and prognostic value of this technique have been demonstrated in different patient groups of the Erlangen Glaucoma registry [16]. Conventional monitors are less suitable for this type of BY-VEP due to low luminance, low refresh rate, and broad emission spectra of the channels. More recently developed stimulators use the micro-mirror-devices incorporated in modern projectors with light emitting diodes (LEDs) as light sources. An advantage of this method is the high temporal resolution (owing to the simultaneous repositioning of the mirrors), narrow band emission spectra of the LEDs, and the high achievable contrast and luminance [13, 17].

The purpose of the present investigation was to investigate whether a projector-based VEP-system can be used to record VEP in healthy participants and glaucoma patients. Here, transient responses of pattern appearance (onset) and disappearance (isoluminant offset) of a blue checkerboard pattern on a yellow background were measured. The diagnostic value of the response amplitudes and peak times and the correlations with conventional perimetrical losses and structural defects, as determined with the SOCT, were studied.

## Methods

### Stimulus

**Fig. 1:**
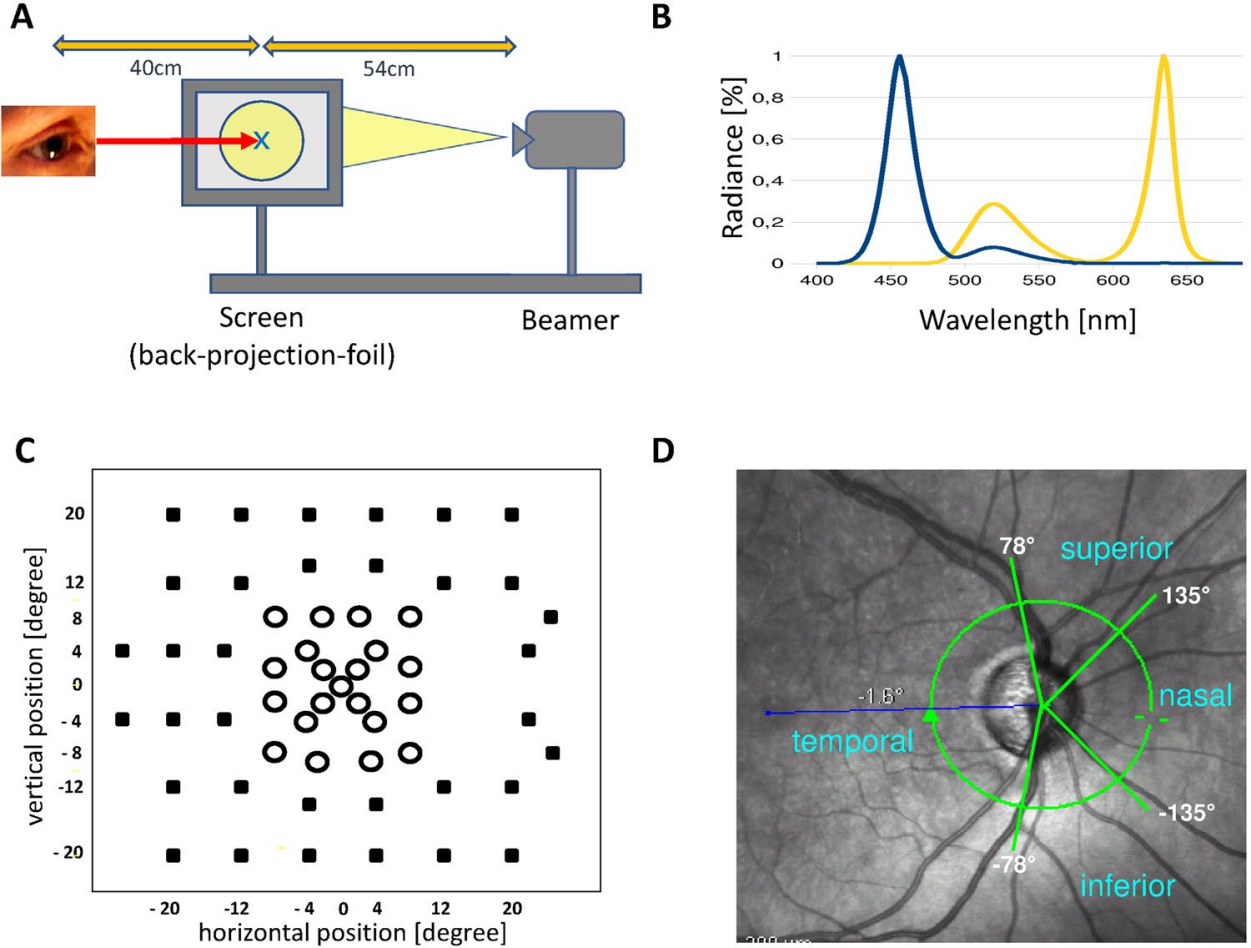
**A** Measurement setting for rear projection with an LED-beamer. **B** Spectra for blue and yellow stimulation by the present beamer. **C** Stimulus locations of the test grid from the Octopus perimeter (G1-protocol). The 21 central test points (open symbols) overlapping with the VEP-stimulus are used for the calculation of the central MD and central SLV. **D** Optic disc segmentation: 4 peripapillary sectors in which the RNFL thickness was determined. The temporal optic disc sectors anatomically overlapped with the VEP test field. The ring indicates the position of the scan circle of the OCT

Projector stimulation and recording of the VEPs were controlled by a modified RETIscan system (Roland Consult, Brandenburg, Germany). The stimulus was presented on a semi-transparent back projection foil (0.32 mm, gain factor 2.0, transparent gray color). The projection foil with its stimulus area was positioned 40 cm from the patient’s eye and 54 cm from the LED-projector (Fig. 1A). The participants were instructed to fixate a cross (0.5° size) in the middle of the checkerboard (0.84 cycles per degree). The measurements were made with natural pupils. To ensure steady fixation we used a chinrest and the participant’s forehead rested against a headband. The experimenter continuously observed the participant and ensured proper fixation. For BYVEP measurements, all participants had appropriate correction lenses for the viewing distance. In addition, before testing with the BY-VEP started, we showed a test-card to the participants to verify the appropriate near correction and gave the participants a preview of the stimulus pattern. All participants reported that they clearly saw the appearance and disappearance of the checkerboard.

The stimulus was created by a single-chip DLP® projector (K11, ACER, Japan) with micro-mirror technology (XGA-mode, 60 Hz). A possible inhomogeneity of the stimulus field can be due to spatial deviation of the projector-illumination or due to the gain-factor of the projection foil that shows an association with the angle of fixation [18]. In the present stimulus the maximal deviation from the mean yellow luminance was –37% (blue luminance of –16%) at a 20 cm distance from the center of the foil measured with a spectroradiometer (Instrument Systems Model CAS 140 CT-151). A circular diaphragm with a 7.3 cm radius was stuck onto the foil to minimize the deviation. As a result, the radius of the test field was 10.3°. Spectral analysis at six positions in the resulting stimulus field on the projection foil did not show any significant deviations in emission spectra or luminance.

The stimuli were presented in a 200 ms pattern onset, 400 ms pattern offset sequence. In the pattern onset phase, the red and green channels of the LED-projector generated a strong yellow (dominant wavelength 574 nm; 619 cd/m^2^ measured with a Tektronix J16/J6503 digital photometer) adaptation light to suppress L- and M-cone sensitivity. During the test, the luminance of the red and green channels of the projector did not change. The blue channel (dominant wavelength 460 nm; 43 cd/m^2^) of the projector was used to superimpose a checkerboard pattern for predominant stimulation of the S-cones (Fig. 1B). We calculated that the spatial Michelson contrast between the checks resulted into 86, 11.5, and 6.4% S-, M-, and L-cone contrast, respectively. Rod contrast was 27% but rod driven responses are expected to be absent due to the high luminance. In the pattern offset period (400 ms) the blue channel generated a homogeneous light with the same time and space averaged luminance as the checkerboard (i.e., 21 cd/m^2^) that was superimposed upon the static yellow background. Thus, the measurements were performed without a change in space and time averaged luminance (640 cd/m^2^). This procedure resulted in a stimulus presentation that was comparable with earlier measurements using an optical bench [12]. The experimental room was dimly lit.

### Recording of the blue-on-yellow VEP

Using the RetiPort system (Electrophysiological diagnostic system Roland Consult, Brandenburg, Germany) VEP recordings were acquired using gold cup skin electrodes (ø 6–10 mm) placed on the participant’s scalp. The ISCEV guidelines were taken as guidance. The active electrode was placed 1 cm above the inion, the reference electrode placed on the forehead approximately 1 cm under the hairline, and the ground electrode was placed on the left earlobe [19, 20]. The skin was cleaned with 96% ethanol and then gently rubbed with skin preparation gel (Nuprep; Weaver and Company, Aurora, CO). The electrodes were filled with electrode paste (TEN Conductive and Adhesive Paste, GE Medical Systems Information Technologies, Freiburg, Germany). The electrodes were then fixated with a headband and one eye covered with an eyepatch; hence the measurements were performed monocularly. The impedances between the electrodes were less than 10 kOhm. Biosignals were band-passed filtered with 1–100 Hz cut-off frequencies and digitized with the appropriate sampling frequency (1700 Hz). In addition, a digital offline filter was applied to the averaged traces to reduce electromagnetic interference from mains. All measurements were repeated three times in each eye (number of averaged sweeps 60–100 per measurement).

**Fig. 2:**
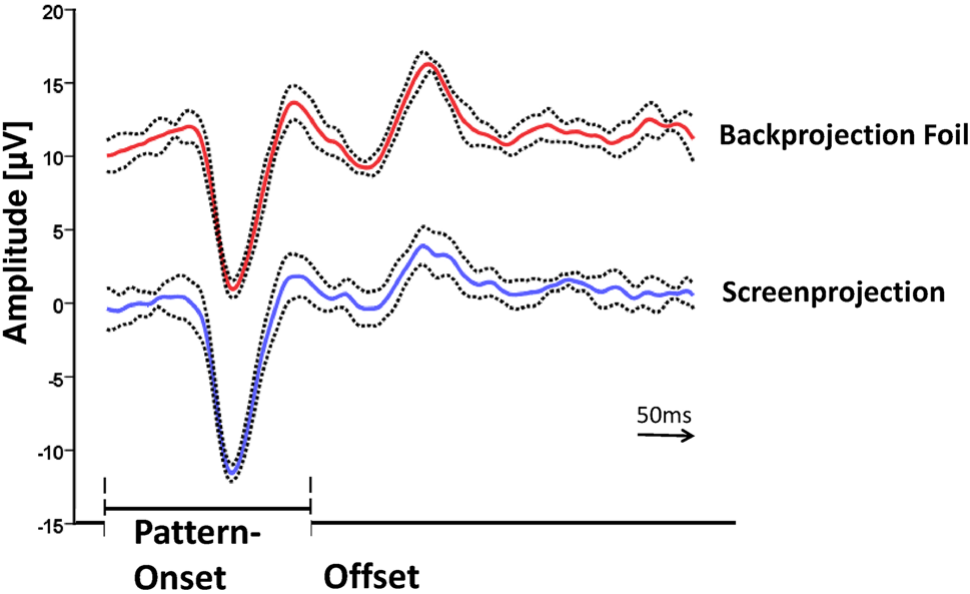
Blue-on-yellow VEP measurements in a healthy participant with the projector comparing on-screen and back-screen projection. In on-screen stimulation, the position of the projector was between patient and the screen. The angle of field size, the spatial frequency, and the luminance was the same for both methods. The dotted lines indicate the 95% confidence interval for six repeated tests

To give an impression of the results using the projector in conventional on-screen mode in comparison to back-screen stimulation, measurements with similar conditions were performed with the same recording setup in a normal participant. The mean of six VEP recordings as elicited with on-screen and back-screen stimulation is presented in Fig. 2. Generally, the traces are very similar to both projection methods and show a small variability for the negative component in reaction to pattern onset. The confidence intervals indicate that the recordings were highly reproducible.

### Study participants

The study followed the tenets of the Declaration of Helsinki for research involving human participants. The study has been registered at www.clinicaltrials.gov (NCT00494923) and was approved by the Local Ethics Committee of the University Hospital. Written informed consent, including agreement for data collection, was obtained from all participants after explanation of the nature and possible consequences of the study. All controls and patients underwent full ophthalmological examinations, including slit lamp inspection, applanation tonometry, fundoscopy (Heidelberg Retinal Tomograph (HRT)), gonioscopy, papillometry, white-on-white perimetry (Octopus automated perimeter O900; Haag-Streit, Schlieren, Switzerland, program G1, 3 phases).

The study included a total of 126 participants (Table 1) of the Erlangen Glaucoma registry [21]. The Erlangen Glaucoma registry (founded in 1991) is a clinical registry for cross-sectional and longitudinal observation of patients with open angle glaucoma or glaucoma suspect. All patients in this study had several earlier examinations in the registry and were experienced in ophthalmological measurements and sensory tests. The mean number of yearly full examination was 15. Due to organizational reasons only patients between 40 and 80 participated in the present study. This is the interesting age range for studying glaucomatous effects. Criteria for the diagnosis of glaucoma were an open anterior chamber angle and classification of glaucomatous changes of the optic nerve head, according to the stages given by Jonas et al. [22]. If both eyes of a participant revealed glaucoma, the eye with lower RNFL was selected. If cataract surgery was performed (20/126, 15.6%) the aphakic eye was selected.

**Table 1:** Descriptive statistic of cohorts. Mean and SD

Groups (number)	age (Years)	SOCT mean RNFL (µm)	MD (dB)	MD central (dB)	SLV (dB)	SLV central (dB)	refractiv error (Diopter)	Visual acuity
Normal (42)	60.5 ± 10.5	96.6 ± 10.3	0.6 ± 1.5	0.7 ± 1.4	1.9 ± 0.5	1.7 ± 0.6	-0.2 ± 2.4	0.9 ± 0.1
OHT (12)	59.2 ± 9.1	92.8 ± 4.6	0.2 ± 1.6	0.2 ± 1.7	1.6 ± 0.5	1.4 ± 0.2	-1.6 ± 3.1	0.9 ± 0.1
Prep. OAG (17)	63.6 ± 8.8	77.1 ± 11.2	0.3 ± 1.3	0.5 ± 1.4	1.9 ± 0.6	1.6 ± 0.7	-1.6 ± 2.4	0.9 ± 0.1
Perim. OAG (55)	66.3 ± 9.4	60.8 ± 12.9	6.3 ± 4.2	6.2 ± 4.6	5.5 ± 2.3	5.6 ± 3.0	–0.8 ± 2.4	0.8 ± 0.2

Seventeen “preperimetric” glaucoma patients (7 females, 10 males, 63.6 ± 8.8 years) had mild damage of the optic disc (stage 1, according to Jonas et al. [22]) and normal visual fields in Octopus perimetry. Fourteen of these patients had elevated intraocular pressure and three had “preperimetric” normal tension glaucoma.

The cohort of “perimetric” open-angle glaucoma patients included 55 patients with primary open-angle glaucoma (33 females, 22 males, 66.3 ± 9.4 years) characterized by glaucomatous optic disc damage and perimetric defects. Forty-four of these patients had elevated intraocular pressure and 11 had normal tension glaucoma.

An OHT-group consisted of 12 patients with intraocular hypertension (6 females, 6 males, 59.2 ± 9.1 years) and normal visual fields and optic disks. For the diagnosis of intraocular hypertension all intraocular pressure measurements were higher than 21 mmHg without medication at two earlier visits. If both eyes from an OHT-patient fulfilled all inclusion criteria, one randomly selected eye was used for statistical analyses.

Forty-two healthy control participants (30 females, 12 males, 60.5 ± 10.5 years) were compared to three cohorts with increasingly severe pathology. All statistical comparisons and correlations used one eye from these control participants. If both eyes of a normal participant fulfilled all inclusion criteria, one eye was selected randomly. In addition, the results of the control participants were used for age normalizing the VEP data. For the age correction the averaged data from right and left eyes were used when both eyes fulfilled the inclusion criteria (in total 36 normal participants). For the remaining six normal participants, the results of only one eye were included in the age correction. This procedure is illustrated in Fig. 4.

None of the participants had any ophthalmological disease other than glaucoma. To exclude color-anomalies, all participants were examined with the Farnsworth D-15 test and an anamaloscope (Nagel and/or Oculus HMC). Furthermore, the majority of the patients (92%) underwent measurements with the Farnsworth FM 100 hue test and/or with the Interzeag color vision meter to uncover blue–yellow anomalies. All individuals revealed clear optic media, a visual acuity of 0.6 or higher, no myopic refractive error > 7 D, and open anterior chamber angle.

### Data analysis

**Fig. 3:**
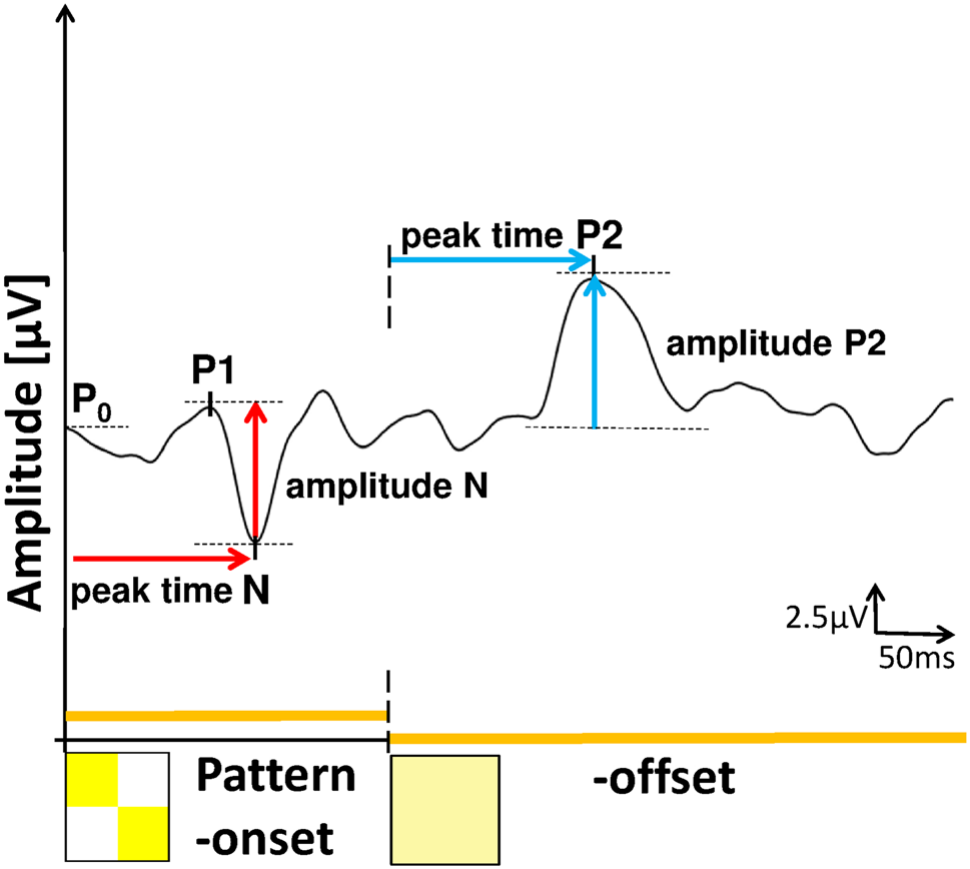
The response to a blue-on-yellow pattern-onset/offset stimulus (yellow light 571 nm, superimposed blue pattern 460 nm). The figure shows the definitions of peak times and amplitudes for the (negative) onset-component and (positive) offset response. The mean luminance was 640 cd/m^2^

From the recorded waveforms the amplitudes and peak times of the following parameters were obtained (Fig. 3 from a normal participant): a negativity (N) during stimulus onset (appearance of the pattern) and a positivity (P2) after pattern offset. The amplitudes of the two components are defined as the difference between N and preceding peak P1 (amplitude N) and the difference between P2 and start-level (P0). For correlation analyses between perimetric measurements and VEP-data only central test locations were considered (21 central test points of the perimetric test routine Octopus G1 (Fig. 1C), total number of test locations, 59). Spectral-domain OCT (Spectralis, Heidelberg Engineering, Germany) images were used to measure the RNFL-thickness along a circle of 3.4 mm diameter around the optic disc. An online tracking system compensated for eye movements during 20–25 consecutive B-scans. Averaged B-scans were analyzed automatically by the Spectralis software to determine the RNFL thickness at 768 positions on the scan circle (Fig. 1D). In the Spectralis software, all retinal vessels within the RNFL were considered to be part of the RNFL. Before the thickness data were exported, the upper and lower border of the RNFL were manually controlled by an experienced assistant. A detailed description of Spectralis-SOCT technique and the analysis can be found elsewhere [23]. To assess the relationship between RNFL-thickness and VEP results, four peripapillary optic disc sectors were defined, as shown in Fig. 1D. The temporal optic disc sector (between –78° and 78°) anatomically overlapped with the VEP test field.

### Statistical Methods

All statistical analyses were performed using SPSS 21 (SPSS-Inc., Chicago, USA). The graphical presentations of the results display mean and 95% confidence interval. The level of significance was alpha = 0.05. Associations between VEP-findings and RNFL values and visual field defects were examined using Spearman’s correlation coefficient. Receiver-operating characteristic (ROC) curves and the area under the ROC curves (AUROC) were used to describe the diagnostic performance of the measurements. Comparisons of groups used ANOVA and Bonferroni post-hoc-tests.

## Results

**Fig. 4:**
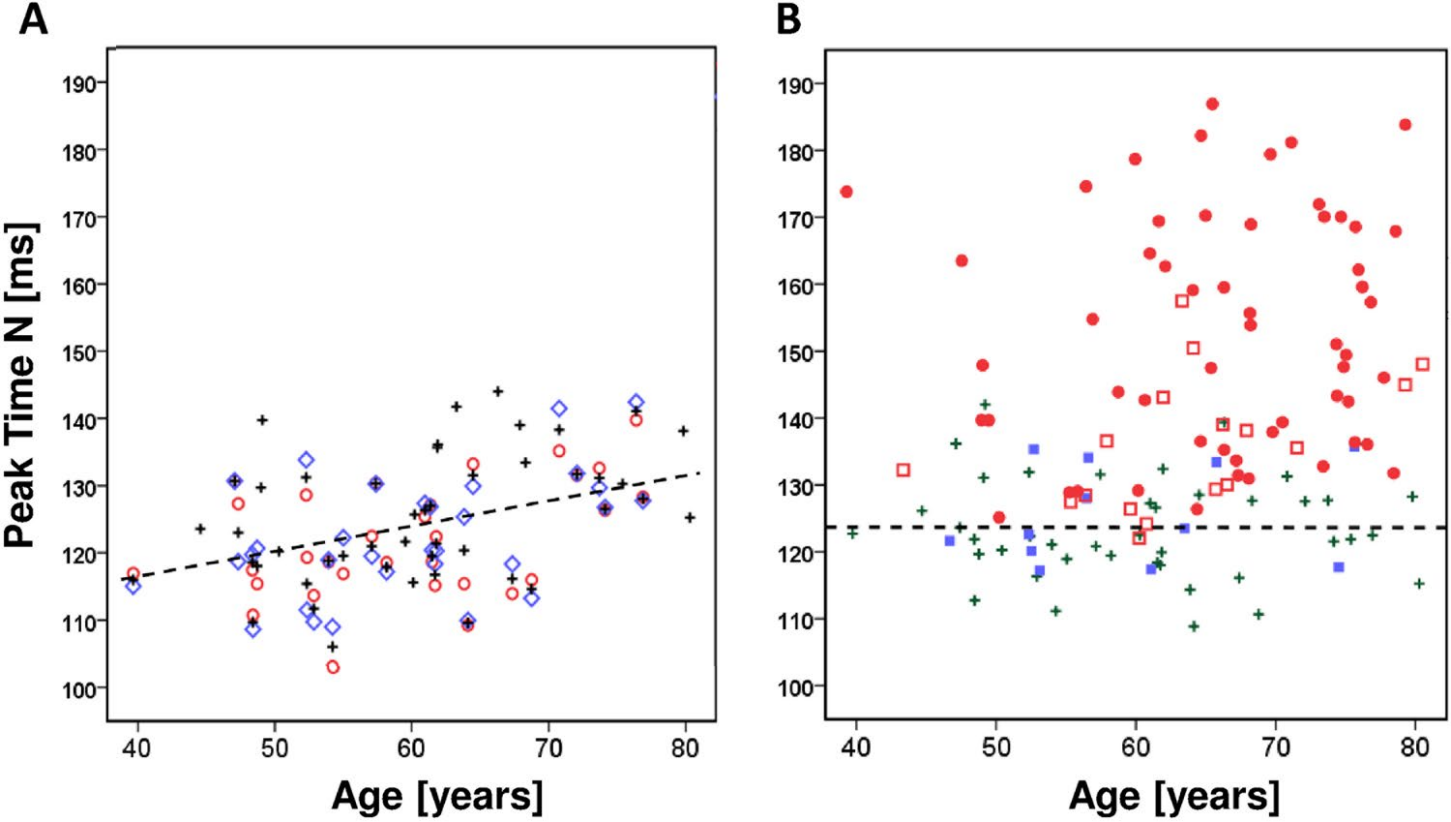
**A** Age-dependence of the peak time of the N component in normal participants. If both eyes were studied (open symbols above and below a cross symbol), the mean from right and left eye were calculated (cross symbol). **B** The data in all groups were age corrected using the regression line as shown in Fig. 4A. Symbols: normal, cross; OHT, filled box; preperimetric glaucoma, open box; perimetric glaucoma, filled circle

As found previously [3, 13, 24], the components of the BY-VEP show an age dependency in the control group (Fig. 4A). In order to reduce the possible influence of age on the comparisons, all amplitudes and peak times of the present BY-VEP measurements were age-normalized by dividing each individual value by the equation of the linear regression of the variable to age and multiplying by the mean. Figure 4B illustrates this procedure for peak time N data in the present participant groups.

**Fig. 5:**
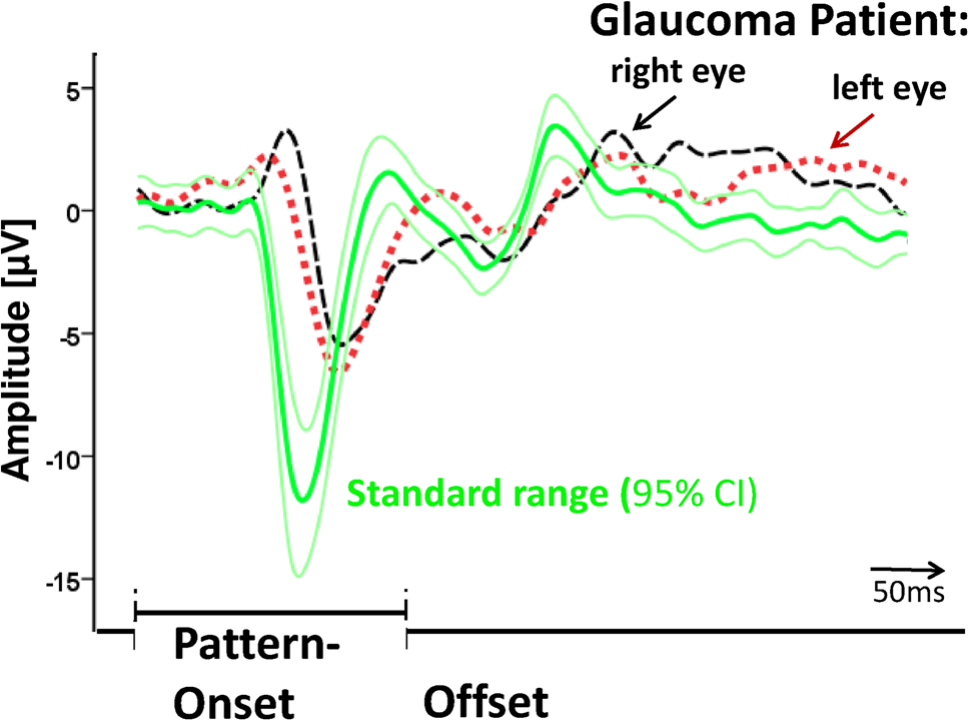
The averaged BY-VEPs (solid line) from ten normal participants (age group 50–60 years). The thin lines indicate 95% confidence interval. Dotted and dashed line show results from a glaucoma patient (age 59 years) with perimetric defects in both eyes

Figure 5 shows BY-VEPs for one glaucoma patient compared to the averaged response of the ten healthy control participants of similar age (age span from 50 to 60 years). This comparison illustrates the peak time prolongation and amplitude reduction of the two response components compared to the normal group. As found in control participants, the pattern-onset generates a negative deflection of the VEP and the offset-response is a positive component [12] in this glaucoma patient.

**Fig. 6:**
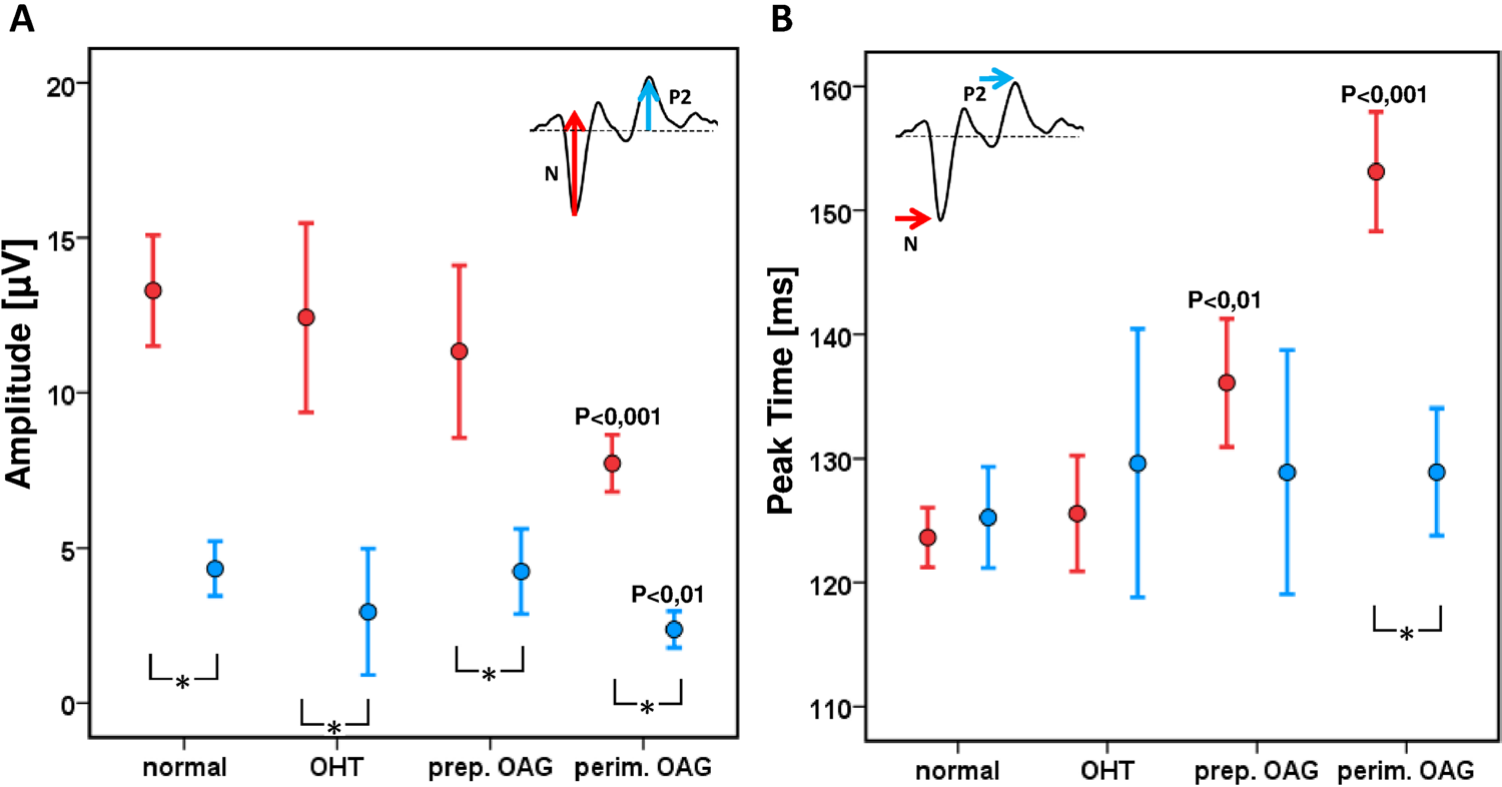
Mean values (± 95 confidence interval) of BY-VEPs in normal participants and three patient groups. **A** Amplitudes. **B** Peak times. *P* values indicate significant differences between patients and control group. Paired comparison of on and offset components with the Wilcoxon-test (*p < 0.001) reveal significant larger onset than offset amplitudes in all groups. Onset and offset peak times differed significantly (*p < 0.001) only in perimetric patients

To analyze the results in all patient groups, all data were age corrected as described above and compared statistically (ANOVA, Bonferroni post-hoc-test). Figure 6A shows the mean values of the amplitudes N and P. The amplitudes decrease with increasing severe glaucomatous changes (*p* < 0.01). The amplitudes in the perimetric patients’ group were significantly smaller compared to the amplitudes of the other groups. The amplitudes measured in OHT- and preperimetric patients did not differ significantly from those found in normal control participants.

Figure 6B displays the peak times of the N and P components for all cohorts. The most striking difference between patients and normal participants can be seen for the negative onset component (N). This peak time is significantly prolonged not only in advanced glaucoma (*p* < 0.001) but also in patients with preperimetric glaucoma (*p* < 0.01). The offset response was not significantly different between the normal participants and the patient groups. The onset time to trough in the “preperimetric” glaucoma group (136.1 ± 10 ms) was significantly prolonged compared to the time in the normal control participants (123.6 ± 7.7 ms) and shorter than the time to trough in the “perimetric” glaucoma patients (153.1 ± 17.8 ms).

**Fig. 7:**
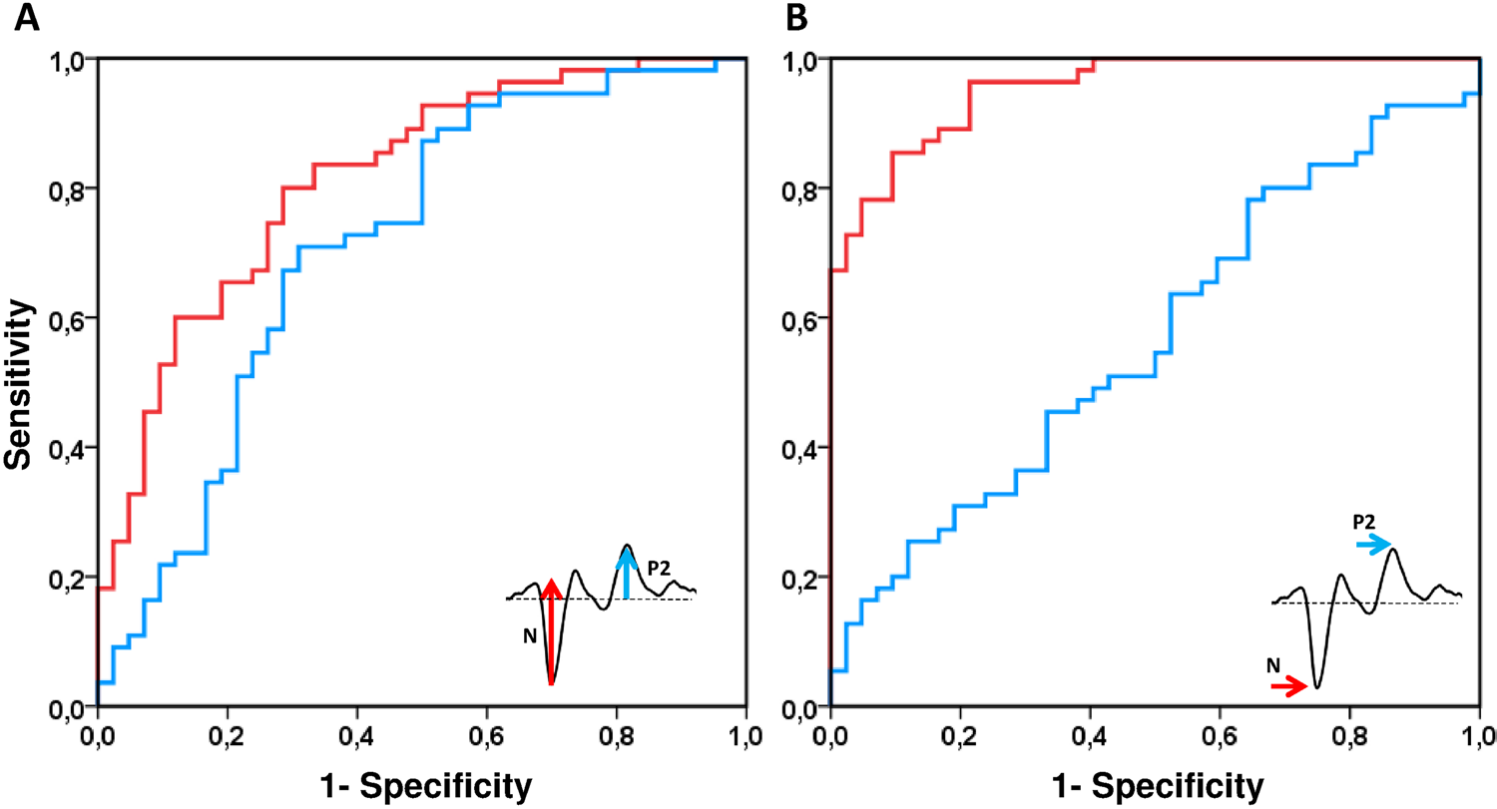
ROC-curves generated for perimetric patients. **A** amplitudes of on- and off-set VEP. **B** Peak times of the (negative) onset-component show the largest diagnostic value

The diagnostic value of the BY-VEP can be judged from the ROC curves of the amplitudes (Fig. 7A) and peak times (Fig. 7B). The ROC curve of amplitudes shows similar specificities and sensitivities for the on- and offset component. The areas under the curves (AUROCs) of the amplitudes were 82% for the onset and 71% for the offset component. The peak time of the negative onset component (N) shows the largest diagnostic value with an AUROC of 95% compared to an AUROC of 57% for the offset peak time P2.

Correlation analyses between amplitude, peak time, and parameters determining the severity of glaucomatous damage revealed significant associations (Spearman rho, *p* < 0.001) for the onset-VEP. Here, the peak time of N is of utmost importance and shows the biggest significance.

**Fig. 8:**
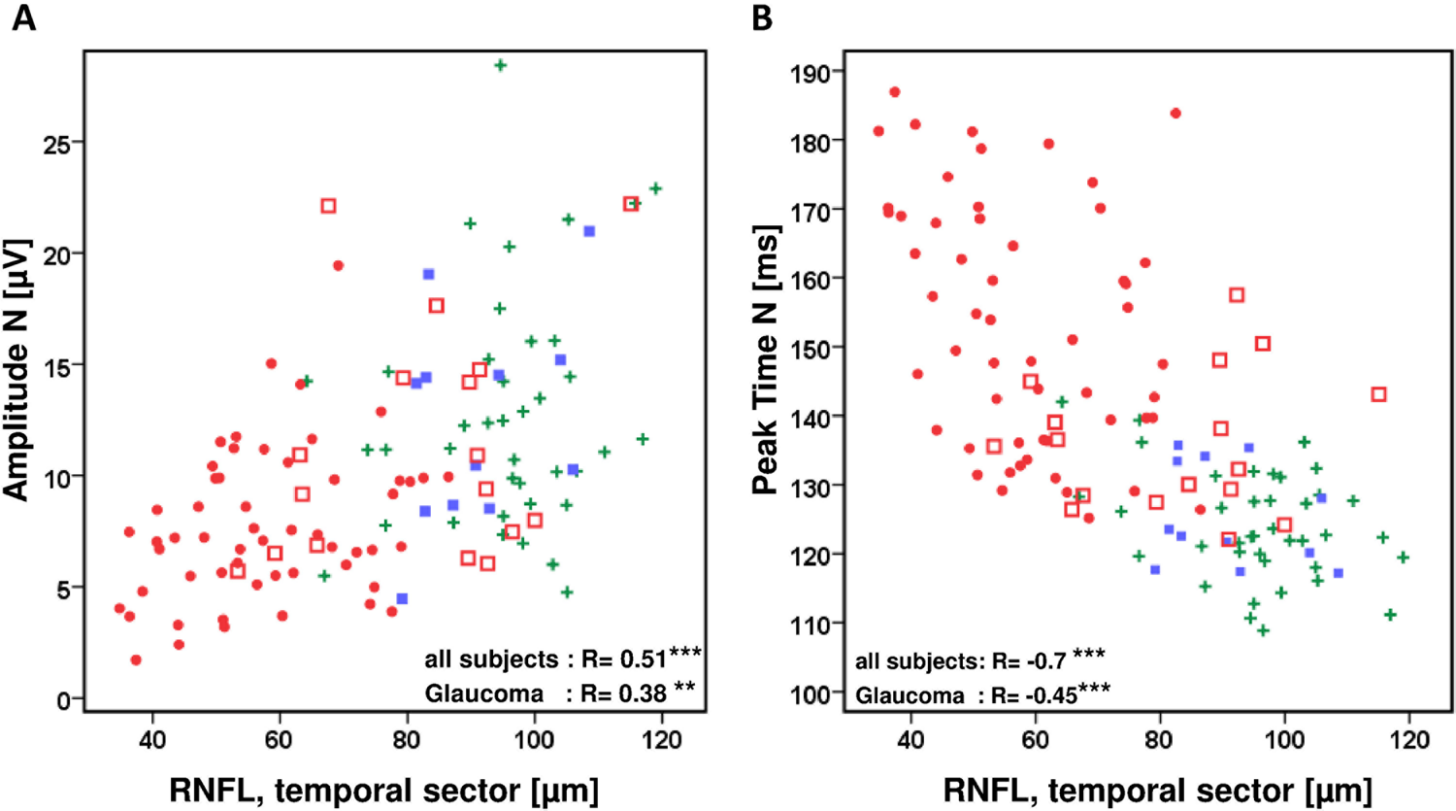
VEP-onset results (**A** amplitude N, **B** peak time N) as a function of thickness of the corresponding RNFL show significant associations. Symbols: normal, cross; OHT, filled box; preperimetric glaucoma, open box; perimetric glaucoma, filled circle. In control participants, the RNFL is thicker, the amplitudes are larger, and the peak times are shorter than in patients. Spearman correlation coefficients are included for all participants and for glaucoma patients only (***p < 0.001, **p < 0.01)

This is shown for RNFL measurements with the SOCT in Fig. 8A for the temporal RNFL sector (amplitudes N R_temporal_ = 0.51) and Fig. 8b (peak times N R_temporal_ = –0.7). The plots show that participants with a thicker temporal optic nerve layer have larger amplitudes in the VEP and shorter peak times in the onset response than patients with reduced RNFL thickness. When the total cohort is considered, significant results are found not only for the temporal sector but also for the correlation with the RNFL thickness in the other sectors, albeit to a lesser extent (amplitudes: R_inferior_ = 0.41, R_superior_ = 0.33, R_nasal_ = 0.26 and peak times: R_inferior_ = –0.65, R_superior_ = –0.56, R_nasal_ = –0.53). When only glaucoma patients are considered in these analyses, the correlations are significant for the temporal sector (Fig. 8) but not for the inferior, superior or nasal sector.

**Fig. 9:**
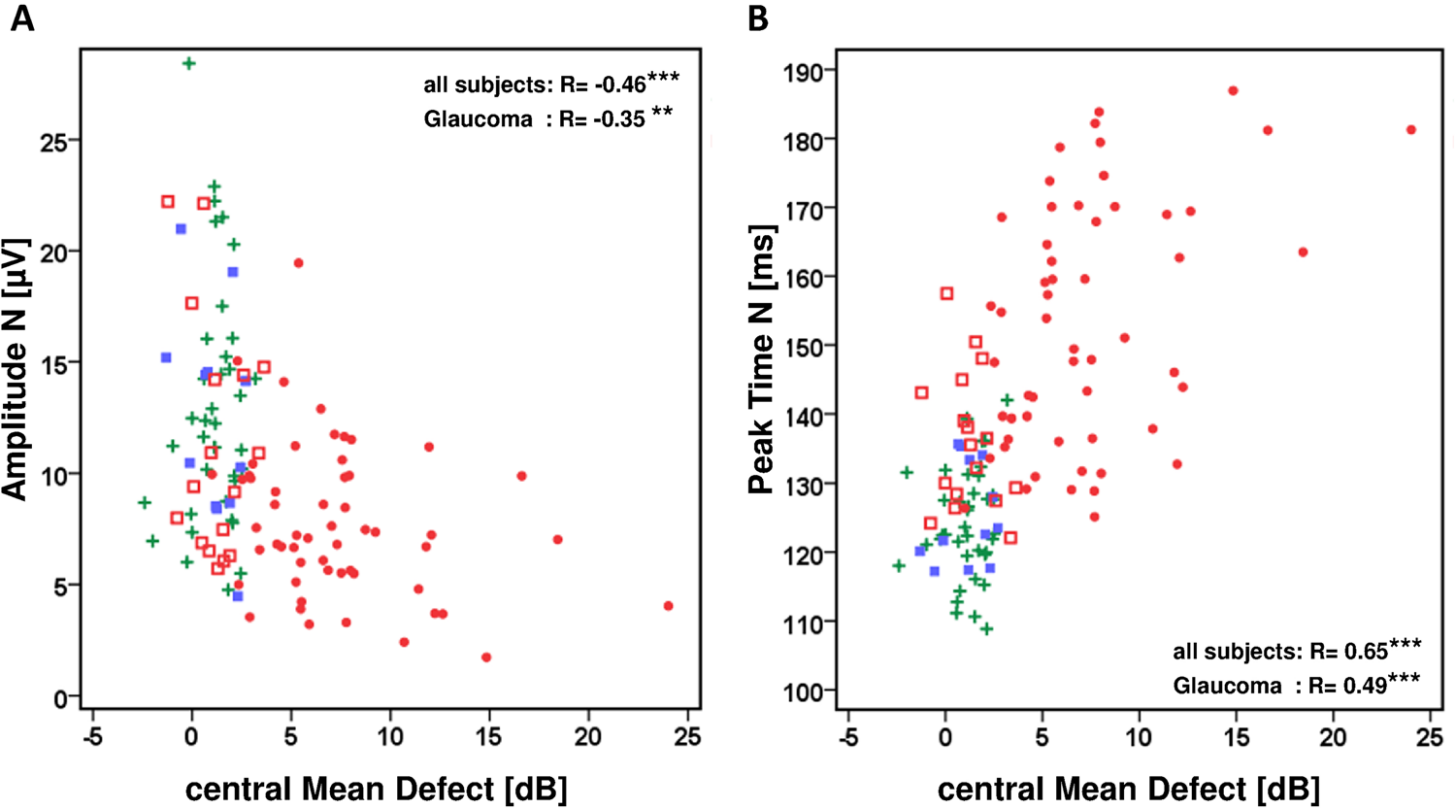
VEP-onset results as a function of central perimetric mean defects (Spearman rho, ***p < 0.001, **p < 0.01). Symbols: normal, cross; OHT, filled box; preperimetric glaucoma, open box; perimetric glaucoma, filled circle

Similar results can be seen when correlation analyses were studied with perimetric losses in the stimulated central retina (amplitudes R = –0.46, peak times R = 0.65). For this analysis only 21 central test points of the perimetric test routine (Octopus G1) were used. The scatterplots in Fig. 9 demonstrate a reduction of the amplitudes and an increase of the peak times in patients with central perimetric losses.

## Discussion

### The blue-on-yellow pattern onset offset-VEP

In the present study, we tried to create stimulus conditions that closely resembled those used in the previous study that employed monochromator filters and a mechanically vibrating mirror in a Maxwellian view system. In contrast to this earlier technique, the modern projector can be easily used in clinical practice. The VEP responses obtained with the Maxwellian view system in earlier studies [3] [12, 13, 16] and the responses obtained with the present LED-projector are very similar.

The aim of this study was to investigate whether the projector technique can be used to elicit reliable BYVEP responses in healthy and glaucoma patients. The present findings with the BYVEP supports the idea that preferential activation of a specific visual channel, in this case the blue–yellow sensitive pathway, is helpful in early glaucoma diagnosis [12, 13] [25–27] because the responses are significantly associated with RNFL thickness and perimetric defects. Earlier studies have shown that glaucoma can reduce the amplitude and increase the peak latency of the BY-VEP [3, 28, 29]. Klistorner et al. used blue–yellow mfVEP to detect early glaucoma and found that amplitudes reveal high sensitive and specificity [26]. Korth et al. [12] introduced a blue-on-yellow technique and found significant associations between VEP amplitudes and peak times on the one hand and perimetry and area of the neuroretinal rim on the other hand. This earlier observation could be confirmed in the present onset-measurements with the projector stimulation. Furthermore, we found a strong diagnostic value of the projector-VEP in early glaucomatous atrophy [12, 13] [3, 30].

In contrast to the negative onset-component, the positive offset-component showed less difference between normal and glaucoma patients and less relationship with glaucomatous damage. The high susceptibility of the BY-VEP to glaucomatous changes is possibly caused by the low number of ganglion cells in this pathway (6% of all ganglion cells) and by a high vulnerability of blue–yellow antagonistic retinal neurons [12] [31, 32]. The possible mechanisms leading to changes in the on- and off-components of the blue-on-yellow VEP have been discussed thoroughly in earlier studies [12, 30]. A drawback of blue–yellow stimulation is that VEP-results are possibly influenced by cataracts [33] in the elderly participants. To minimize the influence of lens opacities on the VEP-results, all parameters were age normalized, eyes with visual acuity exceeding 0.6 were excluded, and the aphacic eye was chosen in participants that underwent cataract surgery. BYVEPs of patients with artificial lenses showed results that were very similar to those of patients with natural lenses.

### Projector stimulation in electrophysiological measurements

The projector as a stimulator can be helpful as a tool for electrophysiological recordings. In contrast to LCD panels and CRTs, which use raster scan mechanisms [34], the digital micro-mirrors of a digital light processing (DLP) projector refreshes all pixels simultaneously and can therefore be used with large temporal precision. In comparison to a monitor that needs 17 ms (at 60 Hz framerate) to create an image, the beamer needs less than 1 ms. In a CRT monitor, each pixel lights up for a few milliseconds each frame and then decays. Nowadays, CRT screens haves been widely replaced as visual stimulators by LCD screens [35]. The pixels of LCD screens have a constant luminance during the duration of the frame, but lights up with a delay [36, 37]. The crystal molecules take a few milliseconds to change their alignment [38]. An advantage of the projector to generate a stimulus is the possibility of back-projection, as used in the present study. Spatial distance between patient and projector minimizes electromagnetic interferences or acoustic disturbances. Furthermore, back-projection and central position of eye and projector helps to reduce possible inhomogeneity of the stimulus [18] [39]. One of the main advantages is that this type of image generation is able to present bright images with high color fidelity and stability [40] [41]. The beamer used in our setup was able to generate a mean luminance = 640 cd/m^2^ in the back-projection mode. If the projector technique is to become a useful test in glaucoma research, it should be directly compared with other modern stimulation techniques [38]. Generally, stimulators cannot simply be exchanged for another without considering that spatiotemporal differences in display technologies and stimuli may affect various facts in experiments [42–44]. Prior to clinical application, the establishment of guidelines for the use of a projector is recommended. Further research and investigation on these more recent technologies may enable us to use BYVEP with projector stimulation in the diagnosis and management of glaucoma.

## Conclusions

Projector-stimulation is a feasible technique for electrophysiological tests and is a useful method for activation of the blue–yellow sensitive pathway because of high luminance and build-up speed. Amplitudes and peak times of the pattern onset VEP show high association with visual field defects and retinal nerve fiber layer thickness.

## Data Availability

The results presented in the manuscript are part of the dissertation by Laura Dussan Molinos (German title: “Blau-auf-gelb VEP mit Beamer-Stimulation als objektive Untersuchung bei Glaukomen”).
